# A combination of omega-3 and plant sterols regulate glucose and lipid metabolism in individuals with impaired glucose regulation: a randomized and controlled clinical trial

**DOI:** 10.1186/s12944-019-1048-x

**Published:** 2019-05-01

**Authors:** Ji-fang Wang, Hai-ming Zhang, Yan-yan Li, Song Xia, Yin Wei, Ling Yang, Dong Wang, Jing-jing Ye, Hao-xiang Li, Jing Yuan, Rui-rong Pan

**Affiliations:** 1 Division of Endocrinology, Affiliated Hospital of Jiangsu University, Zhenjiang, Jiangsu 212000 China; 2 Department of General Surgery, Affiliated Hospital of Jiangsu University, Zhenjiang, Jiangsu 212000 China; 3 Department of Clinical Nutrition, Affiliated Hospital of Jiangsu University, No. 438 Jiefang Road, Zhenjiang District, Jiangsu 212000 China

**Keywords:** Plant sterols, Omega-3 fatty acids, Impaired glucose regulation, Factorial design

## Abstract

**Background:**

Lipid metabolism imbalance has been recognized as one of the major drivers of impaired glucose metabolism in the context of type 2 diabetes mellitus (T2DM), the rates of which are steadily increasing worldwide. Impaired glucose regulation (IGR) plays a vital role in the prevention and treatment of T2DM. The goal of this study was to further clarify whether the combination of plant sterols (PS) and omega-3 fatty acids yields any synergistic effect that enhances the prevention and treatment of IGR.

**Methods:**

A total of 200 participants were randomized to receive PS and omega-3 fatty acids (*n* = 50), PS alone (*n* = 50), omega-3 fatty acids alone (*n* = 50), or placebo soy bean powder plus placebo capsules (*n* = 50) for 12 weeks. Patient characteristics including body composition, blood pressure, glucose metabolism (Fasting plasma glucose (FPG), fasting insulin (FINS), glycosylated hemoglobin (HbA1c), Homeostasis Model Assessment of Insulin Resistance (HOMA-IR)), lipid metabolism (TG, TC, HDL-C, LDL-C) and inflammatory factors (Hs-CRP, IL-6) were all monitored in these IGR individuals.

**Results:**

Compared to the placebo group, the group receiving the combined intervention exhibited significantly decreased TG, HDL-C, FBG, HOMA-IR and HbA1c. Omega-3 fatty acids alone were associated with significant reductions in waistline, TG, FBG, HOMA-IR and Hs-CRP. PS alone was only associated with decreased TG and Hs-CRP. No interventions produced significant changes in body weight, BMI, blood pressure, FINS, body fat percentage, visceral fat rating, TC, LDL-C or IL-6.

**Conclusions:**

In summary, this study has demonstrated for the first time that PS, omega-3 fatty acids or the combination thereof significantly improved inflammation, insulin resistance, as well as glucose and lipid metabolism in IGR individuals. These findings may provide a scientific basis for the development of nutritional products incorporating PS and omega-3 fatty acids, and also for the development of nutritional supplement strategies aimed at preventing the development of disease in the IGR population.

## Introduction

As many societies are undergoing rapid economic development, changes in diet, and transitioning to a sedentary lifestyle, rates of type 2 diabetes mellitus (T2DM) are gradually increasing and as such the disease has become a significant public health concern worldwide. The International Diabetes Federation (IDF) has predicted that diabetes incidence will rise from 366 million in 2011 to 552 million in 2030 [1]. In Asia, the prevalence of diabetes is expected to further increase over the next 20 years. According to a 2010 survey conducted by professor Ning Guang of Ruijin Hospital Shanghai Jiaotong Medical School, the prevalence of T2DM is as high as 11.6% [2].

As known as impaired glucose regulation (IGR), abnormal glucose metabolism can be further broken down into three categories, including impaired fasting glucose (IFG), impaired glucose tolerance (IGT), and IFG complicated by IGT (IFG/IGT). IGR belongs to an intermediate state between normal glucose metabolism (NGT) and T2DM, a state commonly referred to as pre-diabetes. Epidemiological data have shown that IGR patients exhibit multiple risk factors for coronary artery disease, such as insulin resistance (IR), central obesity, hypertension, and high triglyceride (TG) levels [2]. In addition, a long-term prospective clinical study with a large sample size has found that the risk of death from cardiovascular disease or coronary heart disease in IGR patients is about two times greater than in those with normal glucose tolerance, and the overall risk of death is one point five times higher than in those with normal glucose tolerance [3]. It has been clearly shown that IGR is highly reversible, and it is possible to maintain an IGR state or even reverse diabetes in some individuals. IGR and interventions to remediate this state are therefore of major scientific interest, with positive intervention studies being performed in those with IGR generally being carried out to achieve decreased blood glucose, improved glucose tolerance, and to ultimately prevent diabetes progression.

It has been confirmed that patients that have reached the IGR stage already exhibit insulin resistance and insulin secretion defects. Clinical studies strongly suggest that abnormal lipid metabolism is the root cause of glucose metabolism disturbance in T2DM [4]. For example, lipometabolic disturbances induce insulin resistance in peripheral tissues, cellular dysfunction, apoptosis, and necrosis, thus contributing to the progression of diabetes. Waistline size and blood lipid content were both risk factors for abnormal glycometabolic status [5]. A study of serum lipids in rural populations with diabetes and pre-diabetes in Chengdu, China, has suggested that participants with IGR have increased levels of low-density lipoprotein cholesterol (LDL-C) and TG, as well as decreased levels of high-density lipoprotein cholesterol (HDL-C) [6].

In addition, there is ample evidence from epidemiological studies suggesting that chronic inflammation plays a substantial role in the development and progression of insulin resistance, which is defined as the homeostasis model assessment of insulin resistance (HOMA-IR) [7, 8]. As a sensitive index of chronic subclinical inflammation, high-sensitivity C-reactive protein (Hs-CRP) may predict the development of T2DM [9].

Many studies have shown that plant sterols and omega-3 fatty acids are able to regulate lipid metabolism and inflammation [10]. PS, which are not synthesized by the human body, are minimally absorbed by the gut [11] and their structures are very similar to those of cholesterol [12]. Previous studies has found that plant sterols decreased cholesterol and LDL-C. Omega-3 fatty acids decreased VLDL synthesis, while they simultaneously increase lipoprotein lipase expression and fatty acid oxidation [13]. In addition, omega-3 fatty acids have been shown inhibit inflammatory reactions [14, 15]. We therefore speculated that plant sterols and omega-3 fatty acids together might act to improve T2DM or IGR via reducing lipids and inflammatory factors. As this has not previously been studied, we designed a randomized, double-blind, placebo-controlled, 2 × 2 factorial design trial to focus on roles of PS, omega-3 fatty acids, and the combination thereof in IGR individuals, as well as to further determine whether PS and omega-3 fatty acids have synergistic activity in this context. This study will explore the theoretical scientific foundation for the treatment of IGR and T2DM via dietary supplementation.

## Materials and methods

### Study design

This clinical trial was a double-blind, randomized, placebo-controlled study, performed in the affiliated hospital of Jiangsu University from October 2016 to June 2017. The trial has already got approval by the Ethics Committee of the affiliated hospital of Jiangsu University (JSU2016066), and conducted at the Clinical Department of Endocrinology and Metabolism, Medical University of China. This study was registered in the World Health Organization International Clinical Trials Registry Platform (No. ChiCTR-IOR-17013282).

This study was a 2 × 2 factorial design in which subjects were to consume the intervention foods daily over a period of 12 weeks. Of 134 participants, 69 were women (51%), and the range of age was 51–65 years. Participants were randomly assigned to the test groups as follows:

1. Placebo group: placebo soybean powder (placebo 1) + placebo capsules (placebo 2)
2. Omega-3 fatty acids group: placebo 1 + capsules of 2 g fish oil (bioactive components are 1000 mg EPA and 400 mg DHA)
3. PS group: daily flour with 1.7 g plant sterols + placebo 2
4. PS plus omega-3 fatty acids group: daily flour with 1.7 g plant sterols + capsules of 2 g fish oil (1000 mg EPA + 400 mg DHA)

In order to investigate the effects of the dietary intake of these supplements on the development of IGR disease, diabetes education knowledge, dietary and nutritional status, dietary behaviors, and exercise habits needed to be informed and complied with in IGR individuals. To avoid any influence of dietary changes, recruited participants were received health education weekly in the first month, and completed additional education twice a month in the next 2 months.

### Participants

Criteria for study participation included patients with IGR, of which diagnosis was based on the 2012 American Diabetes Association diagnostic criteria [16]. Exclusion criteria included the taking of antidiabetic medication during the intervention period; acute illness within the past 2 weeks; human immunodeficiency virus infection; hepatitis or other significant liver disease (except macroproteinuria); untreated or inadequately treated clinically significant thyroid disease; anemia; active malignant disease; inborn or acquired bleeding disorder; pregnancy or breastfeeding.

All participants were diagnosed as IGR through an oral glucose tolerance test (OGTT) and written informed consent before study enrollment. What’s more, baseline characteristics were collected by blood tests, such as glucose and lipid metabolism related factors, and inflammatory factors. All included participants received personal nutritional counseling on their dietary regimen for about 1 to 1.5 h, and were instructed to maintain a diet with a nutrient ratio of 50–55% carbohydrates, 10–20% protein and < 35% fat. Importantly, participants were instructed not to take any oral antidiabetic drugs and insulin. Finally, PS and omega-3 fatty acids were randomly distributed to subjects. Participants’ compliance to taking the supplements/placebo was assessed by the quantity of surplus drugs, and subjects showed good adherence (up to 99.97%) in this study. In total, 134 IGR patients aged between 51 and 65 years old were recruited for the study.

### Measurements

#### Assessment of anthropometric variables

Baseline enrollment data and blood samples were collected before initiation of the 12-week intervention phase. Participants were measured both at baseline and at the 12 week endpoint for anthropometric indices including height, weight, waist circumference, systolic and diastolic blood pressure. Dietary habits were collected at these same times. BMI was calculated as weight in kg divided by height in meters squared. Body fat percentage and visceral fat rating were determined based on body composition parameters using a bioelectric impedance analysis (TBF300A human body composition analyzer, TANITA, Japan).

#### Assessment of biochemical variables

Eight milliliter venous blood samples were collected after overnight fasting at baseline and 12 weeks after intervention. FPG was measured on the day of blood collection. 2 h postprandial glucose (2hPG) was also measured after consumption of 75 g glucose dissolved in water. Blood samples were immediately centrifuged at 3500 rpm (KA-1000) for 10 min to separate serum at the 0 and 12 week. Serum lipid profiles, HbA1c, FINS, and inflammatory cytokine were also quantified on the day of blood collection. TC, HDL-C, and LDL-C were detected by ultracentrifugation ALBK. TG, Hs-CRP, and IL-6 were measured by glycerol lipase oxidase (GPO-PAP), particle-enhanced immunophelometry, and double antibody sandwich enzyme-link immunoassay (ELISA) respectively. Moreover, the concentration of FINS was determined by isotope labeling tracer.

### Statistical analyses

Statistical analyses included all patients who completed the 3 week trial period and from whom appropriate materials were obtained. Data are presented as means ± SD. To examine differences in baseline characteristics between groups, we used the analysis of variance (ANOVA) test for differences in means for continuous data and the chi-square test for differences in proportions for categorical variables. A two-factor ANOVA was used to test the main effects of dietary supplementation with plant sterols, omega-3 fatty acids, and interactions between plant sterols and omega-3 fatty acids. Statistical analyses were conducted using the Statistical Package for the Social Sciences (SPSS) version 16 (USA). The significance threshold was set at *P* < 0.05.

## Results

### Participant baseline characteristics

**Fig. 1:**
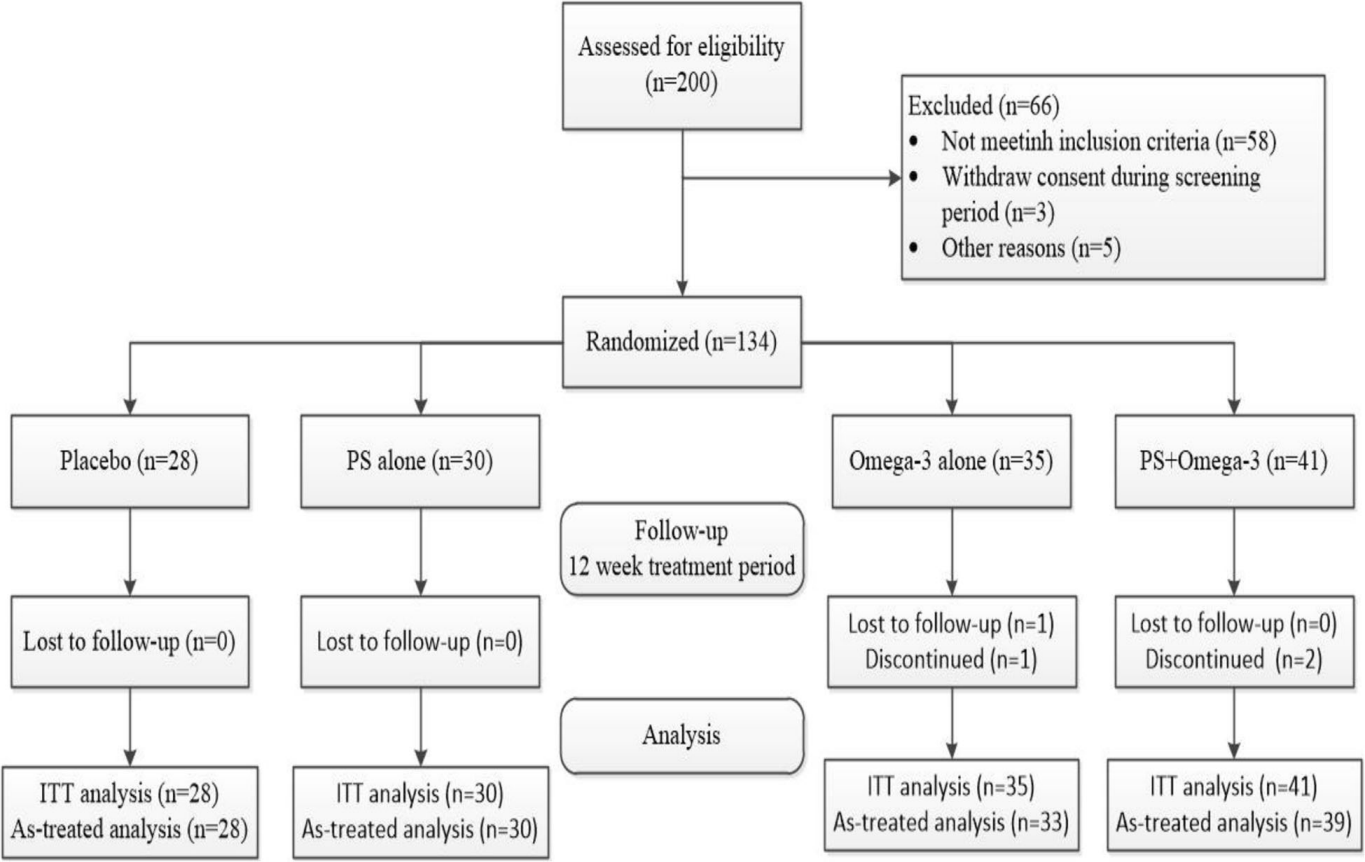
Consort flow diagram. ITT, intent-to-treat

In this study, a total of 134 participants were randomized into the four intervention arms of the trial (Fig. 1). A total of 200 participants were confirmed to meet IGR criteria based on OGTT and were enrolled from October 2016 to June 2017 in the affiliated hospital of Jiangsu University. Of these, 66 participants failed subsequent analyses for reasons including: business limited availability, traffic problem, no acceptance for blood collection, difficulty swallowing capsules, and poor treatment compliance. Table 1 shows the baseline demographic and biomedical characteristics of the study population. Comparative analysis showed no differences in characteristics of body composition, blood pressure, glucose metabolism related parameters, lipid metabolism-related parameters, or inflammatory factors among these four groups.

**Table 1:** Baseline demographic and clinical characteristics

Characteristic	Placebo	PS	Omega-3	PS + Omega-3	*P value*
	*n* = 28	*n* = 30	*n* = 35	*n* = 41	
Female (%)	17 (60.71)	12 (0.40)	17 (48.57)	23 (56.10)	0.39
Age (years)	56.18 ± 4.06	56.17 ± 7.17	58.94 ± 7.21	55.63 ± 7.44	0.16
HT (cm)	162.52 ± 8.72	163.53 ± 8.41	164.23 ± 9.03	162.24 ± 8.06	0.746
Weight (kg)	69.82 ± 10.95	69.52 ± 10.48	69.54 ± 11.81	69.61 ± 10.46	1
BMI (kg/m^2^)	26.45 ± 3.81	26.18 ± 4.71	25.68 ± 2.98	26.43 ± 3.39	0.81
Waistline (cm)	94.84 ± 9.05	90.1 ± 6.71	90.4 ± 8.78	90.85 ± 9.69	0.136
Systolic pressure (mmHg)	132.36 ± 11.81	137.43 ± 12.91	136.69 ± 10.31	138.76 ± 14.57	0.218
Diastolic pressure (mmHg)	84.07 ± 11.15	85.63 ± 9.5	85.91 ± 10.61	84.63 ± 9.3	0.876
FPG (mmol/l)	6.49 ± 0.28	6.44 ± 0.23	6.56 ± 0.27	6.50 ± 0.26	0.369
FINS (mU/L)	10.28 ± 4.13	10.15 ± 3.14	9.85 ± 4.29	10.22 ± 5.47	0.98
HOMA-IR	2.96 ± 1.19	2.9 ± 0.89	2.86 ± 1.21	2.96 ± 1.58	0.983
HbA1c (%)	6.49 ± 0.43	6.21 ± 0.69	6.29 ± 0.74	6.36 ± 0.85	0.487
TC (mmol/l)	5.29 ± 0.82	5.42 ± 0.88	5.39 ± 0.62	5.35 ± 1.16	0.95
TG (mmol/l)	2.05 ± 0.71	2.07 ± 0.94	2.06 ± 0.84	2.07 ± 0.79	0.857
HDL-C (mmol/l)	1.22 ± 0.23	1.14 ± 0.25	1.19 ± 0.17	1.17 ± 0.23	0.632
LDL-C (mmol/l)	3.11 ± 0.90	2.56 ± 0.97	3.00 ± 0.63	2.98 ± 0.80	0.058
Hs-CRP (mg/L)	1.48 ± 0.69	1.13 ± 0.33	1.10 ± 0.85	1.11 ± 0.82	0.13
IL-6 (pg/ml)	3.03 ± 1.19	2.83 ± 1.14	2.98 ± 1.32	2.85 ± 1.36	0.9
Body fat rate (%)	32.68 ± 9.12	29.26 ± 6.97	29.65 ± 7.47	29.87 ± 8.44	0.353
Visceral fat rating	12.07 ± 4.06	11.47 ± 4.41	12.03 ± 4.27	12.07 ± 4.62	0.934

Data were presented as mean ± SD or n (%). *PS* plant sterol, *HT* Height, *BMI* body mass index, *FPG* fasting plasma glucose, *HbA1c* glycated hemoglobin, *TC* total cholesterol, *TG* triglyceride, *HDL-C* high-density lipoprotein cholesterol, *LDL-C* low-density lipoprotein cholesterol, *SD* standard deviation

### Effects on anthropometric measurements

Based on records obtained after the 12-week intervention with PS, omega-3 fatty acids, or the combination of the two, no statistically significant differences were seen between these four groups in terms of weight, body mass index (BMI), body fat percentage, visceral fat rating, systolic pressure, or diastolic pressure. Compared with the placebo group, waistline was decreased in the omega-3 fatty acids group (change from baseline: − 1.90 ± 3.86 vs. -0.88 ± 5.84, *P* < 0.05), while no change was observed in the other two groups (Table 2).

**Table 2:** Effects of plant sterol and Omega-3 supplementation on variables

Variables	Placebo		PS		Omega-3		PS + Omega-3		Main effect of PS	*P value*	Main effect of Omega-3	*P value*	Interaction	*P value*
	Post	Difference	Post	Difference	Post	Difference	Post	Difference						
Weight (kg)	68.97 ± 10.89	−0.85 ± 3.62	68.24 ± 8.14	−1.28 ± 9.14	68.13 ± 11.17	− 1.41 ± 1.91	67.35 ± 10.34	−2.26 ± 2.81	− 0.64	0.47	−0.77	0.38	−0.21	0.81
BMI (kg/m^2^)	26.13 ± 3.8	−0.33 ± 1.33	25.74 ± 4.24	−0.45 ± 3.43	25.19 ± 2.89	−0.50 ± 0.71	25.59 ± 3.55	−0.84 ± 1.02	− 0.23	0.47	− 0.28	0.39	− 0.11	0.73
Waistline (cm)	93.96 ± 9.99	−0.88 ± 5.84	90.77 ± 9.30	0.67 ± 6.04	88.50 ± 9.22	−1.90 ± 3.86	88.88 ± 10.32	−1.97 ± 4.21	0.74	0.40	−1.83	0.04*	−0.81	0.35
Systolic pressure (mmHg)	130.57 ± 13.22	−1.79 ± 14.71	140.23 ± 18.33	2.80 ± 20.50	135.49 ± 12.87	−1.20 ± 16.82	139.44 ± 20.99	0.68 ± 20.85	3.235	0.32	− 0.77	0.81	−1.36	0.68
Diastolic pressure (mmHg)	82.5 ± 12.08	−1.57 ± 7.52	82.33 ± 9.44	−3.3 ± 14.66	79.94 ± 8.86	−5.97 ± 8.23	81.54 ± 10.87	−3.1 ± 8.76	0.57	0.75	−2.10	0.23	2.30	0.19
FPG (mmol/l)	6.43 ± 0.86	−0.06 ± 0.8	6.92 ± 1.01	0.47 ± 0.96	6.34 ± 1.06	−0.21 ± 1.19	5.56 ± 0.62	−0.94 ± 0.66	−0.1	0.55	−0.78	0.01**	−0.63	0.01**
FINS (mU/L)	10.25 ± 3.9	−0.02 ± 3.67	9.57 ± 2.96	−0.58 ± 4.46	9.45 ± 3.87	−0.40 ± 2.25	8.12 ± 3.45	−2.09 ± 2.95	−1.125	0.06	−0.95	0.11	−0.57	0.33
HOMA-IR	2.93 ± 1.17	−0.03 ± 1.04	2.96 ± 1.18	0.06 ± 1.45	2.72 ± 1.34	−0.14 ± 0.70	2.00 ± 0.89	−0.96 ± 0.98	−0.365	0.05*	−0.57	0.00**	−0.46	0.01**
HbA1c (%)	6.35 ± 0.68	−0.15 ± 0.42	6.34 ± 0.92	0.13 ± 1.11	6.15 ± 0.78	−0.14 ± 0.55	6.01 ± 0.73	−0.35 ± 0.51	0.035	0.78	−0.24	0.05*	−0.25	0.05*
TC (mmol/l)	5.23 ± 0.89	−1.73 ± 2.07	5.61 ± 0.95	0.18 ± 1.35	5.05 ± 0.70	−0.33 ± 0.48	4.96 ± 1.16	−0.39 ± 0.87	0.925	0.24	0.42	0.06	−0.99	0.06
TG (mmol/l)	1.82 ± 0.6	−0.23 ± 0.46	1.96 ± 1.27	−0.11 ± 1.32	1.95 ± 0.81	0.16 ± 0.95	1.73 ± 0.64	−0.34 ± 0.77	−0.19	0.01**	0.08	0.61	−0.31	0.01**
HDL-C (mmol/l)	1.10 ± 0.22	−0.12 ± 0.12	1.16 ± 0.19	0.01 ± 0.30	1.21 ± 0.18	0.02 ± 0.19	1.27 ± 0.29	−0.1 ± 0.31	0.005	0.90	0.02	0.73	−0.13	0.05*
LDL-C (mmol/l)	3.02 ± 0.77	−0.09 ± 0.45	2.79 ± 0.78	0.22 ± 1.34	2.92 ± 0.55	−0.08 ± 0.78	2.83 ± 0.88	−0.14 ± 0.75	0.125	0.42	−0.18	0.25	−0.19	0.22
Hs-CRP (mg/L)	1.88 ± 0.53	0.4 ± 0.89	1.08 ± 0.46	−0.05 ± 0.39	0.95 ± 0.43	−0.15 ± 0.59	0.93 ± 0.38	−0.18 ± 0.59	−0.24	0.03*	−0.34	0.03*	0.21	0.06
IL-6 (pg/ml)	3.05 ± 0.87	0.02 ± 1.61	2.49 ± 1.41	−0.33 ± 1.36	2.53 ± 1.37	−0.45 ± 1.7	2.45 ± 1.38	−0.4 ± 1.76	−0.15	0.59	−0.27	0.35	0.20	0.48
Body fat rate (%)	32.28 ± 8.84	−0.39 ± 3.9	28.43 ± 7.13	−0.83 ± 4.74	27.68 ± 5.82	−1.12 ± 4.75	28.11 ± 8.40	−1.76 ± 2.34	−0.54	0.44	−0.83	0.23	−0.1	0.89
Visceral fat rating	11.86 ± 4.49	−0.21 ± 1.55	11.03 ± 4.17	−0.43 ± 3.62	11.14 ± 4.38	−0.83 ± 1.25	11.27 ± 4.49	−0.8 ± 1.47	−0.095	0.79	−0.495	0.19	0.125	0.74

Data were presented as mean ± SD or n (%). *PS* plant sterol, *HT* Height, *BMI* body mass index, *FPG* fasting plasma glucose, *HbA1c* glycated hemoglobin, *TC* total cholesterol, *TG* triglyceride, *HDL-C* high-density lipoprotein cholesterol, *LDL-C* low-density lipoprotein cholesterol, *SD* standard deviation. P* ≤ 0.05; P** ≤ 0.01

### Effects on glucose metabolism factors

In both the omega-3 fatty acid treatment group and the PS plus omega-3 fatty acid treatment group, comparative analysis revealed a significant decrease in the levels of fasting plasma glucose (FPG) (omega-3 fatty acids vs. placebo: − 0.21 ± 1.19 vs. -0.06 ± 0.8, *P* < 0.01; PS plus omega-3 fatty acids vs. placebo: − 0.94 ± 0.66 vs. -0.06 ± 0.8, *P* < 0.01) and HOMA-IR (omega-3 fatty acids vs. placebo: − 0.14 ± 0.70 vs. -0.03 ± 1.04, *P* < 0.01; PS plus omega-3 fatty acids vs. placebo: − 0.96 ± 0.98 vs. -0.03 ± 1.04, *P* < 0.05). In addition, the level of hemoglobin A1c (HbA1c) was decreased upon intervention with PS plus omega-3 fatty acids as compared to placebo (change from baseline: − 0.35 ± 0.51 vs. -0.15 ± 0.42, *P* < 0.05) (Table 2). No interventions were associated with a significant change in fasting insulin (FINS) comparing with placebo. These results therefore demonstrate that PS had no effect on glucose metabolism in IGR individuals, while omega-3 fatty acids and the combination of these two interventions did.

### Effects on lipid metabolism factors

Compared with the placebo group, a decrease in TG concentration was observed in both the PS group (change from baseline: − 0.11 ± 1.32 vs. -0.23 ± 0.46, *P* < 0.01) and the PS plus omega-3 fatty acids group (change from baseline: − 0.34 ± 0.77 vs. -0.23 ± 0.46, *P* < 0.01).

The combination of PS and omega-3 fatty acids also increased HDL-C compared to placebo group (change from baseline: − 0.10 ± 0.31 vs. -0.12 ± 0.12, *P* < 0.05) (Table 2). However, there was no significant effect of either intervention on the concentrations of TC and LDL-C. These findings therefore show that PS and combinatory interventions have positive effects on lipid metabolism in IGR individuals, whereas omega-3 fatty acids alone have no effect.

### Effects on inflammatory cytokines

In intent-to-treat analyses, a decrease in Hs-CRP was observed with both PS (change from baseline: − 0.05 ± 0.39 vs. 0.4 ± 0.89, *P* < 0.05) and omega-3 fatty acids (change from baseline: − 0.15 ± 0.59 vs. 0.4 ± 0.89, *P* < 0.05) (Table 2). However, alteration of Hs-CRP was not observed after the combined intervention of PS plus omega-3 fatty acids. Similarly, there were no statistically significant changes in IL-6 among these four groups. These results suggest that a combined intervention cannot reduce inflammation in the IGR population.

### Interaction of combinatory intervention in IGR individuals

**Fig. 2:**
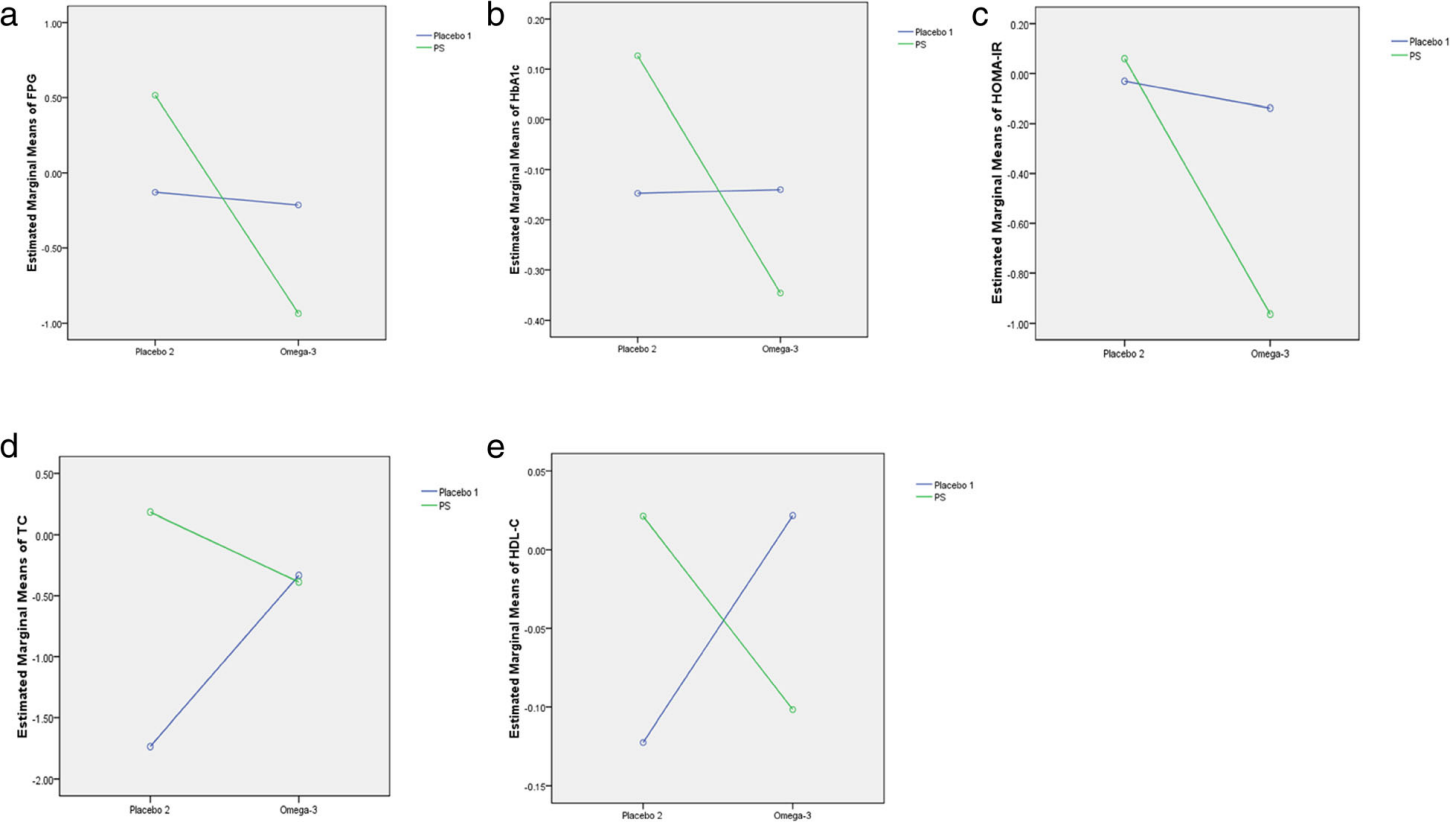
The interaction of PS and omega-3 fatty acids on indicators. **a** interaction diagram of FPG; **b** interaction diagram of HbA1c; **c** interaction diagram of HOMA-IR; **d** interaction diagram of TG; **e** interaction diagram of HDL-C. PS: plant sterol; FPG: fasting plasma glucose; HbA1c: hemoglobin A1c; TG: triglyceride; HDL-C: high-density lipoprotein cholesterol. Data were presented as mean ± SD or n (%). *P* < 0.05 was considered as statistically significant.

Comparative analyses showed that the combinatory intervention facilitated a more significant reduction in FBG and HOMA-IR than either PS or omega-3 fatty acids alone (Fig. 2a, c). These results suggest that PS and omega-3 fatty acids have a synergistic effect on the improvement of insulin resistance and FBG in IGR individuals. Unfortunately, the combined intervention had no synergistic effect on these parameters, such as HDL-C, TG, and HbA1c (Fig. 2b, d, e).

## Discussion

In this randomized clinical trial performed in IGR individuals, we demonstrated that a 12-week treatment with dietary supplements containing PS (daily flour with 1.7 g plant sterols), omega-3 fatty acids, or the combination thereof can significantly alter glucose and lipid metabolism. Specifically, PS predominantly decreased TG and Hs-CRP, whereas omega-3 fatty acids largely reduced HOMA-IR, FBG, waistline size, and Hs-CRP. Overall, these data indicate that dietary interventions, combined or alone, represent an attractive nonpharmacologic strategy for improving metabolic health in IGR individuals.

Plant sterols are comprised of a group of sterols that enter the human body only from dietary sources. Relatively large quantities of plant sterols are present in plant oils, nuts, and avocados. Plant sterols have many important physiological functions; for example, they block the absorption of dietary and endogenously-derived cholesterol in the gut [11]. Researchers have found that plant sterols (0.7–3.2 g/d) can reduce TC by 5.0–13.0%, and LDL-C by 5.6–26.8% from baseline in both normo- and hypercholesterolemic individuals with [17–19] and without [20–24] T2DM. In addition, plant sterols significantly reduced LDL-C in both nondiabetic (by 15.1%) and diabetic (by 26.8%) patients [25]. In a randomized, double-blind, placebo-controlled study [26], plant sterols significantly lowered fasting LDL-C (by 4.6%), TC (by 4.2%), and TG (by 8.3%) in dyslipidemic individuals with or at risk of developing T2DM. In our present study, we have demonstrated for the first time that a significant reduction in TG and Hs-CRP can be achieved in IGR patients via dietary supplementation with plant sterols (2 g/d). Thus, we speculate that PS might prevent IGR progression to T2DM via improving lipid metabolism and inflammation.

Omega-3 fatty acids contain a number of double bonds of unsaturated fatty acid polymers. The most common type of omega-3 fatty acids is alpha-linoleic acid (ALA), which is primarily derived from plant seeds, as well as eicosapentaenoic acid (EPA), and docosahexaenoic acid (DHA), which mainly come from deep sea fish [27]. It has been demonstrated that omega-3 fatty acids can improve peripheral tissue insulin sensitivity by increasing the expression of GLUT4 in the skeletal muscle [28]. Moreover, omega-3 fatty acids also enhance peroxisome proliferator-activated receptor alpha (PPARa) activity, while reducing hepatocyte nuclear factor-4a (HNF-4a) activity. In patients with T2DM, omega-3 fatty acids did not improve glucose metabolism, but high doses reduced the levels of TG and LDL-C [13]. There is ample evidence from clinical trials [26, 29] and animal studies [30] suggesting that omega-3 fatty acids inhibit inflammatory reactions. Therefore, we designed a randomized and placebo-control study to observe whether omega-3 fatty acids can affect inflammatory factors, lipid metabolism, and glucose metabolism in IGR individuals. Our results demonstrated that omega-3 fatty acids improved glucose metabolism, insulin resistance, and inflammation via decreases in FBG, HOMA-IR, waistline size, and Hs-CRP. Our results support studies that indicate omega-3 fatty acids may contribute to IGR treatment.

Espinosa et al. [31] has demonstrated that the combination of omega-3 fatty acids and plant sterols has a positive effect on antioxidant activity. In addition, a study by Bitzur et al. [7] found that treatment with n-3-PSE, which contained 1.3 g omega-3 fatty acids and 1.6 g plant sterols, significantly decreased TG (by 19%) and Hs-CRP (by 7.8%), while there were no significant changes in LDL-C levels. However, there have been only a few studies of the combined interaction of PS and omega-3 fatty acids on diabetic dyslipidemia, and what role the combinatory interaction plays in IGR patients is yet unclear. Our results indicate for the first time that the combinatory interaction of PS and omega-3 fatty acids improves diabetic dyslipidemia, glucose metabolism, and insulin resistance via regulating TG, HDL-C, FBG, HbA1c, and HOMA-IR. In particular, this combinatory interaction had better effects on the improvement of FBG and HOMA-IR than did either single dietary supplement. Interestingly, this combinatory interaction did not decrease waistline size or Hs-CRP, even though omega-3 fatty acids alone significantly decreased these two parameters. This may simply be a consequence of the relatively short observation period utilized in the present study.

In summary, this is the first study to our knowledge that has focused on diabetic dyslipidemia, inflammation, and glucose metabolism by dietary supplementation with PS and omega-3 fatty acids in a randomized, double-blind, placebo-controlled manner in IGR individuals. Based on these results, we believe that this intervention can weaken the inherent lipotoxicity of insulin resistance, promoting positive IGR outcomes. The combined interaction of PS and omega-3 fatty acids, which have therapeutic potential, may offer a safe and effective therapeutic approach in IGR patients. There is a need for additional innovative studies to further investigate inflammatory markers and blood pressure in IGR individuals who take PS and omega-3 fatty acids. For example, the studies need enlarge samples, lengthen observation period, and detect more inflammatory factors. Therefore, PS and omega-3 fatty acids play an important role in the prevention and treatment of IGR progression to T2DM. This study provides support for a scientific approach to using plant sterols and omega-3 fatty acids for the treatment of IGR, as well as T2DM.

N-3 fatty acids decrease TG levels, as well as ameliorate inflammation and endothelial dysfunction [32]. In our study, we haven’t collected N-3 fatty acids levels in participants because of shortage in samples, but beneficial effect of N-3 fatty acids on the metabolic profile of patients with T2DM has been reported. It has been investigated that N-3 fatty acids could decrease cardiometabolic complications of T2DM [33]. Therefore, we speculated that the levels of N-3 fatty acids might be increased in IGR individuals which were received omega-3 fatty acids and the combination of PS and omega-3 fatty acids. In addition, the role and mechanism of N-3 fatty acids on glucose and lipid metabolism in IGR individuals might be a potential research area, also is the direction of our further study.

In summary, we have demonstrated for the first time that omega-3 fatty acids and the combination of PS and omega-3 fatty acids can correct imbalances in glucose and lipid metabolism, and can additionally improve inflammation in IGR individuals. The combined regimen has better efficacy than did either single dietary supplement. This study is of great scientific importance, as it provides a theoretical basis for the diet-based prevention of diabetes and its complications. Nutritional products including PS and omega-3 fatty acids thus have broad application potential for delaying the development of diabetes.

## Abbreviations

FINS: Fasting insulin
FPG: Fasting plasma glucose
HDL-C: High-density lipoprotein cholesterol
HOMA-IR: Homeostasis model assessment of insulin resistance
Hs-CRP: High-sensitivity C-reactive protein
IDF: International Diabetes Federation
IFG: Impaired fasting glucose
IFG/IGT: IFG complicated by IGT
IGR: Impaired glucose regulation
IGT: Impaired glucose tolerance
IR: Insulin resistance
LDL-C: Low-density lipoprotein cholesterol
NGT: Normal glucose metabolism
PS: Plant sterols
T2DM: Type 2 diabetes mellitus
TG: Triglyceride
