# Correction to: A combination of omega-3 and plant sterols regulate glucose and lipid metabolism in individuals with impaired glucose regulation: a randomized and controlled clinical trial

**DOI:** 10.1186/s12944-020-01215-9

**Published:** 2020-03-16

**Authors:** Ji-fang Wang, Hai-ming Zhang, Yan-yan Li, Song Xia, Yin Wei, Ling Yang, Dong Wang, Jing-jing Ye, Hao-xiang Li, Jing Yuan, Rui-rong Pan

**Affiliations:** 1 Division of Endocrinology, Affiliated Hospital of Jiangsu University, Zhenjiang, 212000 Jiangsu China; 2 Department of General Surgery, Affiliated Hospital of Jiangsu University, Zhenjiang, 212000 Jiangsu China; 3 Department of Clinical Nutrition, Affiliated Hospital of Jiangsu University, No. 438 Jiefang Road, Zhenjiang District, 212000 Jiangsu China

**Correction to: Lipids in Health and Disease (2019) 18:106**

**https://doi.org/10.1186/s12944-019-1048-x**

Following publication of the original article [1], the Authors’ Contributions statement need to be changed. It should read as “JFW and RRP conceived of the study and drafted the manuscript. HMZ conceived of the study and revised the manuscript. YYL, SX and GYY participated in the design of the study and performed the statistical analysis. YW, LY, DW, JJY, HXL and JY participated in data curation and helped to draft the manuscript. All authors read and approved the final manuscript.”.
